# Characterization of the Temporomandibular Joint of Southern Sea Otters (*Enhydra lutris nereis*)

**DOI:** 10.3389/fvets.2015.00071

**Published:** 2015-12-09

**Authors:** Danielle Lieske, Natalia Vapniarsky, Frank J. M. Verstraete, Dustin M. Leale, Colleen Young, Boaz Arzi

**Affiliations:** ^1^Department of Surgical and Radiological Sciences, School of Veterinary Medicine, University of California Davis, Davis, CA, USA; ^2^Department of Biomedical Engineering, College of Engineering, University of California Davis, Davis, CA, USA; ^3^Office of Spill Prevention and Response, California Department of Fish and Wildlife, Marine Wildlife Veterinary Care and Research Center, Santa Cruz, CA, USA

**Keywords:** temporomandibular joint, temporomandibular disk, southern sea otter, *Enhydra lutris nereis*, structure–function

## Abstract

The structure–function relationship of the temporomandibular joint (TMJ) of southern sea otter has largely not been described. This study aims to describe the histological, biochemical, and biomechanical features of the TMJ disk in the southern sea otter. The TMJ disks from fresh cadaver heads of southern sea otter adult males (*n* = 8) and females (*n* = 8) acquired from strandings were examined. Following macroscopical evaluation, the TMJs were investigated for their histological, mechanical, and biochemical properties. We found that the sea otter TMJ disks are, in general, similar to other carnivores. Macroscopically, the TMJ disk was highly congruent, and the mandibular head was encased tightly by the mandibular fossa with a thin disk separating the joint into two compartments. Histologically, the articular surfaces were lined with dense fibrous connective tissue that gradually transitioned into one to two cell thick layer of hyaline-like cartilage. The disk fibers were aligned primarily in the rostrocaudal direction and had occasional lacuna with chondrocyte-like cells. The disk was composed primarily of collagen type 1. Biochemical analysis indicates sulfated glycosaminoglycan content lower than other mammals, but significantly higher in male sea otters than female sea otters. Finally, mechanical analysis demonstrated a disk that was not only stronger and stiffer in the rostrocaudal direction than the mediolateral direction but also significantly stronger and stiffer in females than males. We conclude that the congruent design of the TMJ, thin disk, biochemical content, and mechanical properties all reflect a structure–function relationship within the TMJ disk that is likely designed for the sea otter’s hard diet and continuous food intake.

## Introduction

The temporomandibular joint (TMJ) is a synovial joint where the head of the mandible on the condylar process articulates with the mandibular fossa of the temporal bone (1). The fossa and articular surface of the head of the mandible are separated by an articular disk (1). A functioning and healthy TMJ and disk ensures proper occlusion of teeth, effective mastication, stress absorption, and dispersion of masticatory loads (2, 3). In carnivores, the mandibular head moves in a hinge motion, allowing only opening and closing the mouth with a minimal or no lateral movement (4). The TMJ separates mammals from other vertebrates, with its formation intricately associated with the evolution of the three-ossicled ear (5, 6). Among different animal species, the joint morphology, composition, and function vary, reflecting differences in feeding mechanisms, anatomy, and diet (7, 8).

Sea otters (*Enhydra lutris*) are small, blubber-less marine mammals that occupy nearshore habitats in the north Pacific. Lacking blubber, sea otters rely on their dense fur and high metabolic rate for warmth (9, 10). A high metabolic rate necessitates a high rate of food intake; they consume 20–30% of their body weight in food per day (11). Sea otter prey includes a wide variety of benthic invertebrates and fish, though the California subspecies (southern sea otter; *E. lutris nereis*) almost exclusively eats invertebrates, and rarely consumes fish (12). Invertebrate prey species include many hard-shelled animals, such as crabs, sea urchins, mussels, clams, and abalone (11). Although sea otters use tools to open some hard-shelled prey, many prey items are crushed by the teeth and powerful jaws (13). Direct consumption of hard-shelled prey likely correlates with the presence of fractures and attrition seen in otter dentition (11, 14), despite having enamel that is 2.5 times tougher than humans (15). In addition, exposure to abrasive sand particles in the diet may result in dental wear (16). A recent analysis of a large southern sea otter skull collection demonstrated the presence of TMJ osteoarthritis in 4.1% of the specimens (17).

In carnivores, like the sea otters, the caudal end of the condylar process of the mandible takes the form of a transversely oriented cylinder that articulates snugly in a trough-like mandibular fossa (19). Their dental arch includes 32 teeth as an adult consisting of I3/2, C1/1, P3/3, and M1/2 that are dome shaped to dissipate tensile stresses created by high occlusal forces needed for mastication of hard food (14).

Because sea otters must consume ~25% of their body weight a day in food to maintain their high metabolic rate, the presence of TMJ abnormalities may reduce foraging abilities, potentially affecting health and survival. Descriptions of dental anatomy and pathology as well as TMJ disorders for a collection of dry skull specimens of southern sea otters have been reported by our group (14, 17). However, characterization of the soft and hard tissues of the TMJ and understanding of its structure–function relationship was undescribed. Therefore, this study aims to describe the structure–function relationship of the TMJ disk of the southern sea otter based on its morphological, histological, and biochemical properties. Furthermore, our aim is to describe the biomechanical characteristics of healthy TMJ disks.

## Materials and Methods

### Specimens

The heads of 16 (8 females and 8 males) fresh dead or euthanized southern sea otter carcasses were obtained from the California Department of Fish and Wildlife sea otter necropsy program in Santa Cruz, CA, USA, with authorization from the US Fish and Wildlife Service. All of the specimens were skeletally mature adults containing fully erupted adult dentition; five specimens were further classified as aged adults (estimated age ≥10 years). After collection, each head was labeled with age, sex, and a unique identification number, and then the specimens were stored at −20°C for 1–5 months prior to dissection. Prior to dissection, the heads were thawed at 20°C for 18–24 h.

### Gross Evaluation

Cone beam-computed tomography (CBCT) of the otter skulls was performed in order to provide aid and reference for gross evaluation and description of the anatomical features. Transverse images were obtained at a slice thickness of 0.3 mm and the CBCT images were processed using Invivo5 software (Anatomage, San Jose, CA, USA) and evaluated on a medical-grade flat-screen monitor. Three-dimensional reconstructive images were generated to assess the spatial relationship of the bones of the TMJ. The TMJs were evaluated by observation and palpation as well as manipulation of the jaws to evaluate the range of motion and jaws movements.

### Microscopic Evaluation

The TMJ disks were removed and fixed in 10% buffered formalin for 48 h. In addition, the mandibular heads and mandibular fossa of the temporal bones as well as one whole joint were fixed in 10% buffered formalin and underwent additional decalcification in 15% formic acid prior to tissue processing. Samples were then paraffin embedded and sectioned at 5 μm and stained with hematoxylin and eosin (H&E) according to standard protocol. Additionally, 5 μm sections were stained with picrosirius red for collagen content and structure, safranin-O for glycosaminoglycan (GAG) content, and Bielschowsky silver for nerve fibers. Examination under polarized light on the picrosirius red stained slides was performed to assess collagen organization. Standard protocols were followed when producing the special stains. Sections were assessed histologically by a veterinary pathologist (Natalia Vapniarsky).

### Immunohistochemistry Evaluation

Five micrometers formalin-fixed paraffin-embedded sections of the TMJ disk were labeled for collagen type I and type II. The slides were deparaffinized in two consequent washes of xylene and then rehydrated in descending concentrations of ethanol (100, 95, 80, and 75%). The endogenous peroxidases were blocked by 3% H_2_O_2_ in methanol. Subsequently, the samples were blocked with goat or horse serum over 1 h at room temperature. Monoclonal mouse anti-collagen type I (Abcam ab90395) or rabbit anti-collagen type II (Abcam ab34712) primary antibodies were applied and incubated at 4°C overnight. After subsequent washing, secondary antibodies (Vectastain ABC Kit, Vector Labs) were utilized and incubated for 30 min at room temperature. The color was developed with DAB substrate. The tissue sections were counterstained with Weigert’s hematoxylin. Sections of native bovine menisci were used as positive and negative control tissues for collagen type I and collagen type II labeling.

### Biochemical Characterization

Punch biopsy samples (3 mm in diameter and ~0.3 mm in thickness) from the approximate central region of the TMJ disk were used for biochemical analysis. Disk tissue was weighed and measured prior to and following 48 h of lyophilization. The lyophilized tissue was digested in 125 μg/mL papain (Sigma, St. Louis, MO, USA) in phosphate buffer (pH 6.5) containing 2 mM *N*-acetyl cysteine (Sigma) and 2 mM ethylenediaminetetraacetic acid for 18 h at 60°C. GAG was quantified using a Blyscan GAG assay (Bicolor, Westbury, NY, USA) based on 1,9-dimethylmethyl blue binding.

### Mechanical Characterization

Tensile testing was performed on an Instron 5565 (Instron, Norwood, MA, USA). Testing was conducted following American Standardized Testing Materials (ASTM) Standard D3039. Rectangular samples, measuring ~5–15 mm in length, 1.51 ± 0.34 mm in width, and 0.28 ± 0.09 mm in thickness, of the TMJ disk in the rostrocaudal and mediolateral direction were obtained from TMJ disks stored in protease inhibitor solution and evaluated using the Instron (18). Samples were positioned between two clamps and elongated at a rate of 1% of gage length per second and the load–displacement data were used to develop a stress–strain curve. The linear portion of the stress–strain curve was used to determine Young’s modulus (EY) and the ultimate tensile strength experienced by the disk tissue. Image J software (National Institutes of Health, Bethesda, MD, USA) was utilized for the measurement of the cross-sectional width and depth of disk samples.

### Scanning Electron Microscopy

Samples obtained from the joint were fixed in 3% glutaraldehyde for 48 h at 4°C. They were then dehydrated in an ascending series of ethanol. After critical point drying, the samples were sputter coated with gold prior to imaging on a Phenom Pro Desktop SEM (PhenomWorld, Eindhoven, the Netherlands). Collagen fiber quantification and bundle diameter were performed using ImageJ analysis software (NIH).

### Statistical Analysis

To evaluate significant differences in mechanical and biochemical properties of the TMJ disk between male and female southern sea otter, a Student’s *t*-test was used. A *P*-value of <0.05 was considered statistically significant.

## Results

### Gross Evaluation

**Figure 1:**
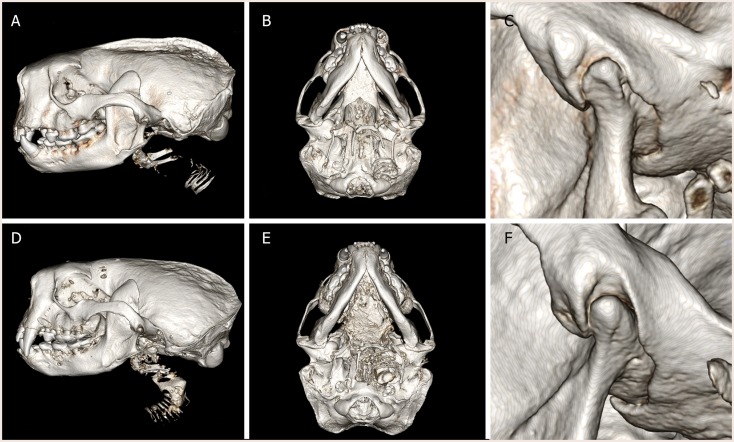
**Three-dimensional reconstruction of computed tomography images of (A–C) male southern sea otter and (D–F) female southern sea otter**. Lateral view **(A,D)**, ventral view **(B,E)**, and close up view of the left TMJ, lateral view **(C,F)**. The condylar process of the mandible is positioned at the occlusal plane. The mandibular fossa of the squamous temporal bone tightly encased the mandibular head on the condylar process.

The spatial position of the TMJ of southern sea otters was found to be at the level of the teeth occlusion (Figure 1). The TMJ was found to be composed of a fusiform-like mandibular head on a condylar process. The articular surfaces were smooth and glistening. The mandibular fossa of the temporal bone engulfed most of the mandibular head. Due to the tight and congruent articulation, it was difficult to separate the mandibular head from the fossa. A disk measuring 0.28 ± 0.09 mm in thickness in the sampled TMJ disks was separating the mandibular head and mandibular fossa.

### Microscopic Evaluation

**Figure 2:**
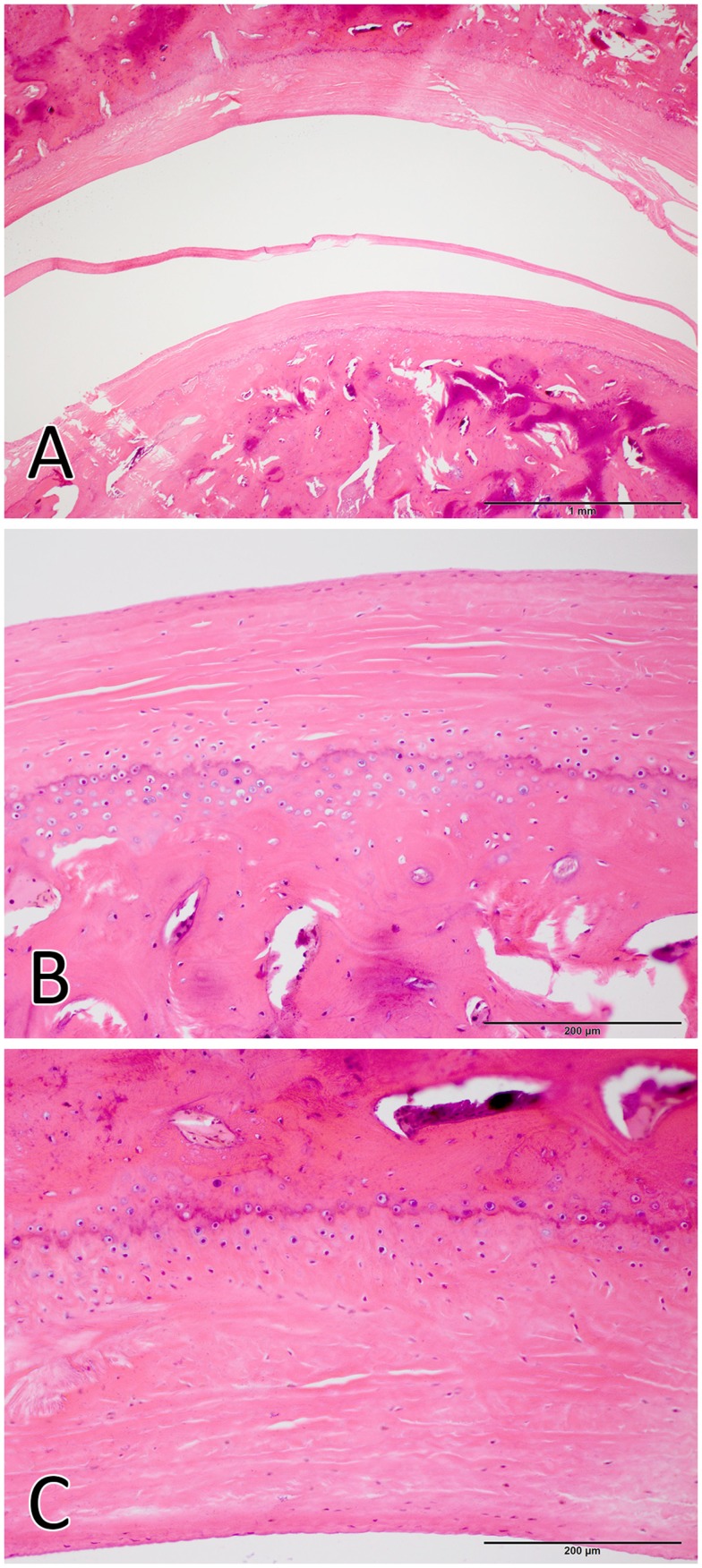
**Histological image of the total joint (A), the mandibular head (B), and the mandibular fossa (C)**. The articular surfaces were lined by ~200 μm layer of dense fibrous connective tissue that gradually transitioned into one to two cell thick layer of hyaline-like cartilage. [H&E staining Bar = 1 mm for **(A)** and Bar = 200 μm for **(B,C)**].

**Figure 3:**
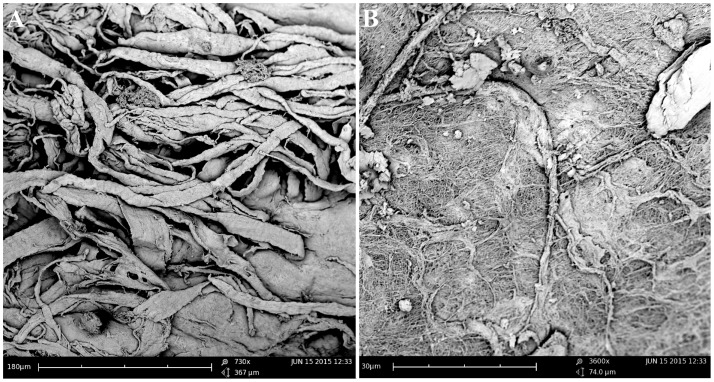
**Scanning electron microscopy of the TMJ disk in cut section (A) and surface (B) views demonstrating that collagen fiber bundles were oriented in orthogonally intersecting planes**. In the center of the disk, the orientation of fibers was primarily rostrocaudal, but in the anterior and posterior parts fiber, orientation was much more anisotropic.

**Figure 4:**
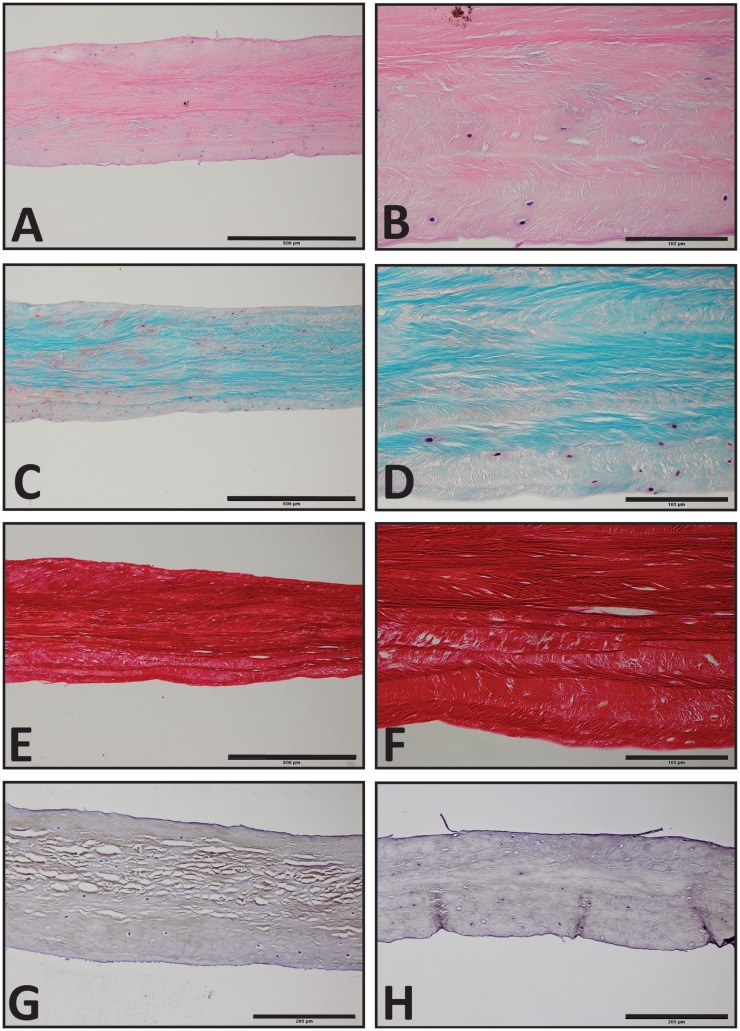
**Histological section of the southern sea otters TMJ disk**. **(A,C,E)** Sea otter TMJ disk H&E, Safranin-O, and Picrosirius red, respectively, 10× scale bar = 500 μm. **(B,D,F)** Sea Otter TMJ disk H&E, Safranin-O, and Picro Sirius Red, respectively, 40× scale bar = 100 μm. **(G)** Sea Otter TMJ disk demonstrates immunoreactivity for collagen type I, 20× scale bar = 200 μm. **(H)** Sea otter TMJ is no immunoreactive for collagen type II, 20× scale bar = 200 μm.

The TMJs of the examined sea otters were found to be similar to other carnivores. Macroscopically, the TMJ was highly congruent with concave mandibular fossa tightly encasing dorsally convex mandibular head of the condylar process. A thin TMJ disk separated the joint into two compartments. Histologically, the mandibular fossa was lined by ~200 μm layer of dense fibrous connective tissue that gradually transitioned into one to two cell thick layer of hyaline-like cartilage outlined by distinct tidemark line, which in turn transitioned immediately into ossified cartilage zone (Figure 2). Within the fibrous layer, rare oval cells resembling small chondrocyte were scattered randomly among rostro-caudally oriented collagen fiber bundles. Similar histomorphology was observed in the section of corresponding mandibular head (Figure 2). A thin, 0.049–0.069 mm, TMJ disk was biconcave and histologically was composed of collagen fiber bundles oriented in orthogonally intersecting planes (Figure 3). In the center of the disk, the orientation of fibers was primarily rostrocaudal, but in the rostral and caudal parts fiber orientation was much more anisotropic. Occasional chondrocyte-like cells residing in tight lacunae were randomly distributed among the collagen fiber bundles. Each lacuna was outlined by minimal amount of hyaline matrix. In the central portions, the disks were avascular but small diameter blood vessels and occasional small islands of adipose cells were present at the periphery of the disk. This observation was supported by safranin-O staining, which highlighted sulfated glycosaminoglycan (sGAG) substance around the lacunae in faint orange (Figure 4). The collagen nature of the disk is highlighted by intensely positive picrosirius red staining. No nerve fibers were observed in H&E or Bielschowsky silver stained sections of the disk.

### Immunohistochemistry Evaluation

Immunohistochemical examination revealed faint but diffuse immunoreactivity for collagen type I and almost complete absence of immunoreactivity for collagen type II (Figure 4). The faint staining for collagen type I may have been due to poor cross-reactivity of monoclonal anti human collagen type I antibody with otter tissue.

### Biochemical Characterization

**Figure 5:**
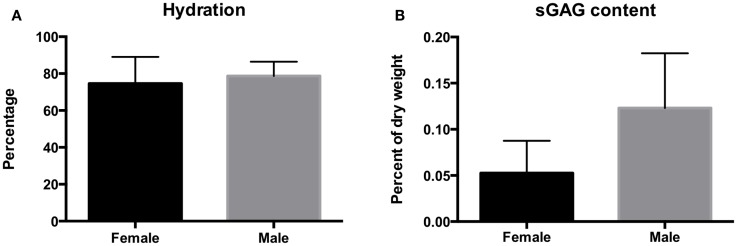
**The hydration (A) and sulfated GAG content (B) of the disks of the southern sea otter**. Hydration averaged 74.6 ± 14.4% per dry weight for female otters and 78.7 ± 7.7% per dry weight for male otters. Sulfated GAG content averaged 0.12 ± 0.06% for the males and 0.05 ± 0.03% for the females.

Biochemical analysis is represented in Figure 5. The hydration of the disks ranged between 56 and 92%. Hydration averaged 74.6 ± 14% dry weight for female otters and 78.5 ± 7.7% dry weight for male otters. There was no significant difference in hydration percentage between males and females (*P* = 0.3999). The GAG content per dry weight on contrary was significantly higher in males than in females and averaged 0.12 ± 0.06 and 0.05 ± 0.03%, respectively (*P* = 0.024).

### Mechanical Characterization

**Figure 6:**
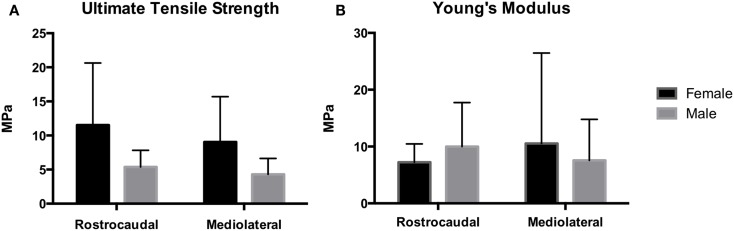
**Mechanical analysis demonstrates Young’s modulus for the female and male southern sea otters (B) averaged 7.23 ± 3.24 and 9.99 ± 7.74 MPa in the rostrocaudal direction, respectively, and 10.5 ± 15.9 and 7.56 ± 7.24 MPa in the mediolateral direction, respectively**. UTS for the female and male southern sea otters **(A)** averaged 11.5 ± 9.1 and 5.38 ± 2.54 MPa in the rostrocaudal direction, respectively, and 9.06 ± 6.64 and 4.30 ± 2.34 MPa in the mediolateral direction, respectively.

Tensile strength of the TMJ disks is demonstrated in Figure 6. Ultimate tensile strength in the rostrocaudal direction for the females and males averaged 11.5 ± 9.1 and 5.38 ± 2.54 MPa, respectively. In the mediolateral direction, tensile strength averaged 9.06 ± 6.64 and 4.30 ± 2.34 MPa in females and males, respectively. There was a significant difference in tensile strength between males and females in the rostrocaudal (*P* = 0.046) and mediolateral (*P* = 0.043) direction. Young’s modulus for the female otters in the rostrocaudal and mediolateral direction averaged 7.23 ± 3.24 and 10.5 ± 15.9 MPa, respectively. The male otters averaged 9.99 ± 7.74 MPa in the rostrocaudal direction and 7.56 ± 7.24 MPa in the mediolateral direction. There was no significant difference between males and females in regard to Young’s modulus in neither the rostrocaudal direction (*P* = 0.5180) nor the mediolateral direction (*P* = 0.6315).

## Discussion

The present study provides the first insight into the structure–function relationship within the TMJ of southern sea otters, as well as important characteristics of the TMJ disk of this subspecies. The TMJ has a tight congruency with the mandibular fossa encasing most of the mandibular head. In addition, the mechanical strength and sulfated GAGs of the disk in females were significantly different than that of males indicating stronger TMJ disk in females.

The TMJ of southern sea otters and its associated soft tissue and bony structures carried similarities to that of other carnivores that rely on heavy mastication (19, 20). Specifically, the mandibular head on the condylar process articulated with the mandibular fossa of the squamous temporal bone (1). The joint was separated to two compartments by a remarkably thin disk. The TMJ was highly congruent and encapsulated tightly by the mandibular fossa. In addition, the spatial location of the TMJ was at the occlusal level as is seen in other carnivores. None of the samples examined had gross or histological signs of joint pathology, which is in agreement with our previous report on low occurrence of TMJ pathology in southern sea otters (17). These findings indicate that the TMJ of southern sea otters is a highly specialized joint adapted to absorb stress and strain with high reliability. Furthermore, the exceptionally thin joint disk may suggest that the majority of the loads are distributed through the large articular surface area of the joint rather than being supported by the disk (18, 21). This TMJ design is likely advantageous as sea otters need to meet a large daily energy requirement. Due to their lack of blubber, sea otters rely on metabolic heat loss from consumption of food items to maintain their body temperature (9, 11, 12). With mass-specific daily energy requirements ranging from 0.57 MJ day^−1^ kg^−1^ for an adult male to 0.87 MJ day^−1^ kg^−1^ for a molting pup, an average 29 kg adult male sea otter would need to consume nearly 4,000 kcal day^−1^ (22, 23). In addition, many sea otters consume a diet of hard-shelled prey, which sea otters must crush to obtain the fleshy, calorically dense portions (11, 12, 24). Therefore, it is possible that the TMJ of southern sea otters is designed to accommodate great masticatory strength with long-term dependability.

Biochemical and mechanical characteristics of the southern sea otter’s TMJ disk suggest functions other than solely supporting tensile and compressive forces. We found that the GAG content was lower than the reported values for California sea lion (25), pig (26, 27), African elephant (28), and human TMJ disks (8). GAG and its associated proteoglycans, such as biglycan and decorin, are typical for fibrocartilage and may play a role in collagen fibril formation and orientation and may contribute to increased tensile strength. The higher concentration of GAGs in male otters was associated with lower tensile strength which somewhat contradict this notion (8, 29). Also, within our specimens, the average ultimate tensile strength and tensile stiffness was higher in the rostrocaudal direction than in the mediolateral direction, as often seen in other species (8). The average ultimate tensile strength and tensile stiffness was lower than those reported in California sea lions in both the rostrocaudal and mediolateral direction (25). This may be due to the differing feeding mechanisms and prey species of otters and sea lions. Sea lions use a pierce-feeding method compared to the chewing of sea otters (25).

Biochemical and mechanical analysis indicated that in addition to species specific adaptations, there may also be differences between males and females. To the best of our knowledge, sex-specific differences have not been described for other species of mustelids or carnivores, and it is not immediately obvious why such a disparity would exist for sea otters. One possible explanation would be consumption of different prey species between male and female sea otters. However, extensive research on sea otter foraging patterns, especially in California, do not support that hypothesis. Research has indicated that sea otters of both sexes specialize in consuming two or three prey items, which comprise the majority of the diet, and individual prey preferences are passed from mother to offspring (30, 31). However, foraging strategies can differ slightly between males and females. For example, Bodkin et al. found that male sea otters made deeper foraging dives than females (32). Despite decades of thorough observations, no studies have indicated any difference in the frequency or abundance of consumption of any prey items between males and females. This observation is also supported by the equal rate of tool-use, which sea otters may use to open hard-shelled prey, by males and females (13). Therefore, a prey-mediated difference in TMJ tensile strength and GAG content between males and females is extremely unlikely.

A behavioral difference between male and female sea otters is the next logical explanation for the observed sex-based differences in TMJ characteristics. Like foraging, a multitude of studies over the last several decades have documented the behavioral characteristics of sea otters. Behavioral research indicates that male and female exhibit an almost entirely identical range of behaviors; however, mating and pup rearing have sex-specific differences, both including differing uses of the mouth/jaw between the sexes. During mating, the male sea otter grasps the female, which often includes the male biting the nose/rostrum of the female, sometimes for extended periods of time (12). Observations indicate that this behavior is more frequent and more aggressive among the male southern sea otters compared to other subspecies of sea otter. Females, on the other hand, use their mouths/jaws for carrying/dragging their pups (11). Females also share food with their pups, which potentially could result in increased food processing (i.e., increased use of the TMJ), however, the combined food intake of a female and her pup is still less than an adult male (22). Since females of many species carry their young in their mouths, it would be worth looking for sex-specific differences in TMJ characteristics in those species as well to determine whether this is the likely explanation for the sex-based differences observed in the sea otter TMJ. Other, currently uninvestigated explanations for the differences we observed also are possible.

In conclusion, the present study provides a unique insight into the structure–function characteristics of the southern sea otter TMJ and provides a comparison between sexes. Our findings suggest that the TMJ structure of the species is adapted to the sea otter’s diet and feeding behavior. The TMJ is specialized to absorb stress and strain with high reliability and that southern sea otters rely more on articulating bony structures then the disk. Males and females differ in the mechanical and biochemical characteristics of the disk that is likely linked to their feeding behavior. We hope that this study will inform on the role of southern sea otter’s TMJ in feeding, function, and potentially for population recovery.

## Author Contributions

DL – acquisition of data, analysis and interpretation of data, drafting of manuscript, critical revision of the manuscript for important intellectual content. NV – acquisition of data, analysis and interpretation of data, critical revision of the manuscript for important intellectual content. FV – study concept and design, critical revision of the manuscript for important intellectual content. DML – acquisition of data, technical and material support. CY – acquisition of specimens, critical revision of the manuscript for important intellectual content. BA – study concept and design, acquisition of data, drafting of manuscript critical revision of the manuscript for important intellectual content – principle investigator.

## Conflict of Interest Statement

The authors declare that the research was conducted in the absence of any commercial or financial relationships that could be construed as a potential conflict of interest.
